# Fruit for Hospitals

**Published:** 1886-10-30

**Authors:** 


### FRUIT FOR HOSPITALS.

People are very good in sending flowers to our hospitals, so we hope, as they hear more about their sick brethren, they will learn that the doctors would allow them to have fruit and green salads too. They will, when orchards or gardens are full, send other delicacies from their abundance. Hampers might at any time be sent to the matrons, to be distributed by the sisters in proper cases ; they would be a great boon. The authorities are always willing to receive ; so too much cannot be sent to our crowded town hospitals.

When some wards were busy or full of serious cases, the fruit, etc., could easily be passed on to others for consumption. If rich people knew how little the poor can taste of these things, and how much appetite and pleasure they give—tone to the system, salines to the blood, help for wounds to heal—they would send in quantities of what they grow too much of for themselves, and not leave it to go to seed, or to drop on the ground, or to be sold at a loss.

Hospital fare includes much that is needful—bread, meat, and milk—but not Nature's wholesome fruit and green-food in abundance. It is true that a very few obtain some of the latter, but others look on with eager eyes, able to eat and make good use of what physicians and surgeons, deterred by the expense, cannot order for them. Large and small hampers might come any day to any hospital, from one or other of the kindly disposed ; and gratitude, and the sight and taste of fruit or fresh green salads, would elevate and cheer many a heart, which now feels forgotten by its more fortunate fellowman. A Hospital Nurse.

